# Indents

**Published:** 1902-08-15

**Authors:** 


### INDENTS.

**☞ Brief Articles of Technical or Scientific Value will receive notice and comment. Contributions invited.**

CLEANLINESS.—Never take a patient into your operating room without cleaning up after the one just dismissed. No matter how busy you are.. Not alone for sanitary reasons, but also for business reasons.—R. O. Williams, *Cosmos.*

TO RECOVER SWALLOWED SUBSTANCES.—If possible to recover by emetic, give the patient some pultaceous food like oat meal to eat before giving the emetic. If it can be passed through the bowels, give the patient plenty of bulky food.—*International Journal of Surgery.*

CALCIUM CHLORID TO PREVENT HEMORRHAGE.—Dr. Wallis, in the *British Medical Journal*, says that he gave calcium clorid three times a day in five grain doses for a week to prepare an hemophilic patient to have several teeth extracted, with the result of very slight hemorrhage.

NEW TEMPORARY STOPPING.—Dr. Barrellier, in the *Revue Odontolologique*, advocates the use of cotton impregnated with a paste made of oxid of zinc by hydrogen dioxid as a temporary dressing. Such stoppings were found in good condition after one week in mouths unhygienically kept.

TO PREVENT MIRRORS FROM CLOUDING.—Slightly moisten the finger and pass it over a cake of soap, and apply to the surface of the mirror; then rub the mirror dry with a clean cloth, and it will be as bright as ever and will not tarnish when put into mouth.—P. F. Soudern, in *New York Medical Journal*.

Mixed Anesthetics.—Dr. Madurs, in the *Medical News*, says that plethoric people take nitrous oxid well, but ether poorly, unless chloroform be given intermittantly. He suggests that in practical anesthesia, nitrous oxid be given first, and this followed by a few inhallations of ether, and then chloroform be given for eight or ten minutes, and that the anesthesia be completed and kept up with ether diluted with a small quantity of air.

Bockin Porcelain Facings.—J. R. Beach, of Clarksville, Tenn., suggests a swaging appliance, made of two pieces of three-quarter inch brass tubing of proper lengths, fitted with guide pins. Both tubes are filled nearly full with modeling compound, into one of which the facing after the backing has been approximately adjusted is imbedded, and the upper section of tubing is placed in correct opposition and forced together by a blow from a suitable hammer.—*Cosmos*.

To Correctly Articulate Gold Crowns.—Fit the band as usual, allowing space for cap, then solder a cap on the band of thin pure gold plate, and cut a good sized hole in its center. Stamp a proper cusp cap and partially fill it with solder and dress to fit the band and articulate with occluding teeth, then tack in position with a little solder. Then place on the root to verify occlusion, remove and complete the soldering through the opening in the pure gold cap.—Dr. Johnson, in *Cosmos*.

Treatment of Pyorrhea.—Dr. R. Good, of Chicago, first washes out the pockets with sterilized warm water. He then anesthetizes the pockets with twelve percent cocain solution (a one percent solution would be sufficiently effective and very much less harmful, ED.). He then thoroughly cleanses the teeth, treating one or two teeth at a time. After the roots and pockets are cleaned, he floods the pockets with chemically pure warmed lactic acid. The object of the lactic acid is to disinfect and start healthy granulations.—*Cosmos*.

Difficult Impressions.—J. V. Barrenechea in the June *Cosmos* describes a method of taking impressions of irregular arches such as are met in getting models for partial plates or regulating devices. He takes a suitable impression tray and saws it into four pieces; once across the front part, and then back along the lateral sides. This is then waxed together with sticky wax and the impression taken in plaster of paris. When the plaster has hardened the front section is removed and this will give the impression at least of the labial and incisal surfaces of the six anterior teeth. The lateral pieces are then removed giving an impression of the buccal and occlusal surfaces of the bicuspids and molars. This leaves the palatial section, to remove which, gives an impression of the palate and lingual surfaces of all the teeth. The sections are then assembled and the model poured. We suppose the same idea could be used for the lower arch, but this would necessitate some modification in cutting the tray.

